# Cardiovascular Health Effects of Internet-Based Encouragements to Do Daily Workplace Stair-Walks: Randomized Controlled Trial

**DOI:** 10.2196/jmir.2340

**Published:** 2013-06-21

**Authors:** Lars Louis Andersen, Emil Sundstrup, Marianne Boysen, Markus Due Jakobsen, Ole Steen Mortensen, Roger Persson

**Affiliations:** ^1^National Research Centre for the Working EnvironmentCopenhagenDenmark; ^2^University Hospital KøgeDepartment of Occupational MedicineKøgeDenmark; ^3^Lund UniversityDepartment of PsychologyLundSweden

**Keywords:** physical activity, Internet technology, sedentary, fitness, vigorous activity, intervention, randomized controlled trial, stair-walk, blood pressure, body mass index

## Abstract

**Background:**

Although the hazardous health effects of a sedentary lifestyle are well known, many adults struggle with regular physical activity. Simple and efficient encouragements for increased physical activity are needed.

**Objective:**

To determine the effect on cardiovascular health of email-based encouragements to do daily stair-walks at work together with colleagues among adults in sedentary occupations.

**Methods:**

A single-blind randomized controlled trial was performed at a large administrative company in Copenhagen, Denmark. Participants were 160 office workers (125 women, 35 men; mean age 42 years, SD 10; sitting 89.5% of work time). At baseline, aerobic fitness was 37 mL/min/kg (SD 9), mean blood pressure was 118/79 mmHg (SD 14/9), and mean body mass index (BMI) was 23 kg/m^2^ (SD 4). Participants were randomly assigned (2:1 ratio) to an email group receiving weekly email-based encouragements to walk the stairs for 10 minutes a day or to a control group receiving weekly reminders to continue their usual physical activities. The primary outcome was the change from baseline to 10-week follow-up in aerobic fitness determined from a maximal cycle test. The examiner was blinded to group allocation.

**Results:**

Adherence to the email encouragements was fairly high with 82.7% of the participants performing at least 3 sessions of 10-minute stair-walks per week (mean 3.3, SD 1.3). Mean heart rate reached 167 beats/min (SD 10) during stair-walks. In the intention-to-treat analysis, aerobic fitness increased 1.45 mL/min/kg (95% CI 0.64-2.27) at 10-week follow-up in the email group compared with the control group. In participants with low aerobic fitness at baseline (n=56), aerobic fitness increased 1.89 mL/min/kg (95% CI 0.53-3.24), and systolic and diastolic blood pressure decreased 4.81 mmHg (95% CI 0.47-9.16) and 2.67 mmHg (95% CI 0.01-5.32), respectively, in the email group compared with the control group. Body weight decreased in the email group of those with low aerobic fitness compared with the control group, but this was not statistically significant.

**Conclusions:**

Simple and inexpensive email-based encouragements to do daily stair-walks together with colleagues at work improves cardiovascular health among adults in sedentary occupations. There exists an enormous potential to prevent the hazardous health effects of a sedentary lifestyle through the use of email-based encouragements to do short bouts of physical activity at the workplace.

**Trial Registration:**

Clinicaltrials.gov NCT01293253; http://clinicaltrials.gov/ct2/show/NCT01293253 (Archived by WebCite at http://www.webcitation.org/6HWG2jw68).

## Introduction

According to the World Health Organization, lack of physical activity is ranked as the fourth leading cause of all deaths worldwide [1]. To promote and maintain health, the American College of Sports Medicine (ACSM) and the American Heart Association (AHA) recommend that healthy working-age adults perform moderate aerobic physical activity for at least 30 minutes on 5 days each week or vigorous aerobic physical activity for at least 20 minutes on 3 days each week [2]. In spite of this knowledge, surveys from North America and Britain show that only one-quarter to one-half of adults self-report that they meet public recommendations for physical activity [3,4]. Thus, lack of sufficient physical activity in the population remains a problem.

The workplace may provide an optimal setting to encourage a healthier lifestyle because most adults spend the majority of their waking hours at work together with colleagues, many with similar needs for physical activity [5]. Furthermore, in modern society most adult workers have the opportunity to communicate and organize physical activities through emails or Internet-based media. In addition, overcoming the motivational barriers for performing regular physical activity is easier in a social setting, such as the workplace, than individually [6]. Internet technology helps create social networks; thus, social media may be used as a community setting at workplaces to encourage a healthier lifestyle and motivate physical activities. However, the need for special facilities and subsequent showering limits the feasibility of prolonged and strenuous physical exercise programs at the workplace. Further, lack of time is often cited as the major reason among adults for not being physically active [7]. Thus, short bouts of vigorous physical activity at the workplace that can easily be organized by using Internet technology and without the need for special facilities and subsequent showering may be preferred [8]. Robroek and coworkers [9] showed that employees receiving monthly email prompts were 6 times more likely than those not receiving email prompts to continue using an Internet-based physical activity and healthy nutrition program at the workplace. Thus, emails and Internet-based media may be an efficient and inexpensive method to organize and motivate participation in such physical activities.

Stair-walking is a vigorous form of physical activity requiring 5 to 10 times the energy expenditure of rest [10]. As most office buildings have accessible stairways, stair-walking may be a feasible form of physical activity at the workplace that can be encouraged and organized using Internet technology. Most stair-walk interventions at the workplace have reported positive effects on aerobic fitness, blood pressure, and/or blood lipids [11-15], which is important from a preventive perspective because a high level of aerobic fitness is associated with decreased risk for cardiovascular and all-cause mortality [16,17]. However, methodological limitations were present in many of the previous stair-walk studies, for example, no control group or no blinding of examiners. Further, none of the aforementioned studies used Internet technology as part of the intervention. Thus, high-quality randomized controlled trials investigating the health effects of Internet-based encouragements to do daily physical activity at the workplace are needed. Companies play an important role in health promotion, but randomized studies have failed to show positive return of investment of health-promoting activities at the workplace probably because the cost of interventions are often high [18]. Consequently, as many companies have limited resources for health-promoting activities, the implementation must be simple and inexpensive. Email-based encouragements are cheap, easy to administer, and have the ability to reach virtually all employees at office workplaces; thus, they may result in mutual benefit for employees and employers. One option could be to send out emails to encourage using the stairs as part of daily routine, for example, when having meetings on another floor, and using the stairs instead of elevators [14]. Encouragements could also be delivered through emails to form groups with colleagues and do planned stair-walking as an active break to supplement overall physical activity. The present study uses this approach.

The aim of the present study is to determine the effect of email-based encouragement to do daily stair-walks together with colleagues on cardiovascular health among adults in sedentary occupations. It was hypothesized that email-based encouragements to do daily stair-walking improves aerobic fitness (primary outcome).

## Methods

### Ethical Approval and Trial Registration

The Local Ethical Committee approved the study protocol (H-3-2010-062). The trial was registered prior to enrollment of participants (Clinicaltrials.gov NCT01293253), which ensured that the study aim, hypothesis, and primary outcome were predefined. There were no changes in outcomes after the trial commenced. All participants gave written informed consent in agreement with the Declaration of Helsinki.

### Study Design and Flow of Participants

**Figure 1:**
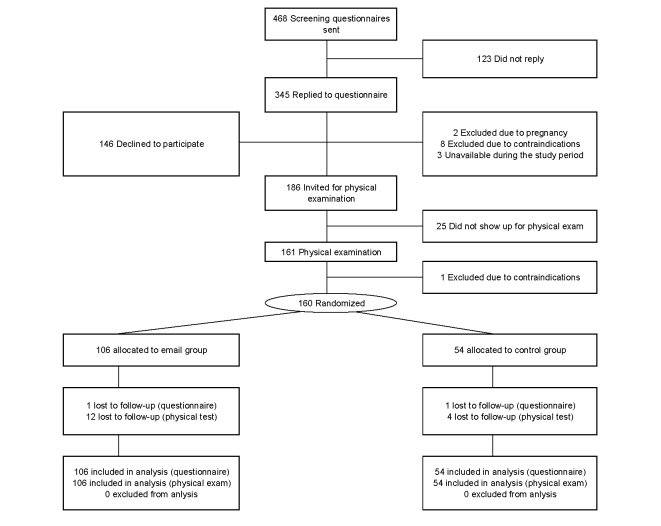
CONSORT flow diagram of participants through the study.

A randomized controlled trial was performed in Copenhagen, Denmark, from February to June 2011. The CONSORT-EHEALTH checklist was followed to ensure transparent and standardized reporting of the trial [19]. Figure 1 shows the flow of participants through the trial. A screening questionnaire on health, physical activity, and working conditions went out by email to 468 office workers in a large administrative company (ie, a single workplace), and 345 replied. All participants had computer/Internet literacy. The inclusion criterion were willingness to participate in the study (N=199). Exclusion criteria were (1) a medical history of life-threatening disease (n=8), (2) current pregnancy (n=2), (3) unavailable during the study period (n=3), and (4) blood pressure greater than 160/100 mmHg (determined later during physical examination). An invitation for a physical examination was sent to the remaining 186 employees, and 161 showed up. During the physical examination, 1 employee was excluded because of blood pressure greater than 160/100 mmHg. Thus, the final sample size was 160 participants. A blinded examiner measured blood pressure and aerobic fitness of the employees at baseline and at 10-week follow-up as described subsequently.

### Randomization and Blinding

Using a computer-generated random numbers table, the 160 participants were randomly allocated to the email group or control group (2:1 ratio) following the baseline examination. Simple randomization was used. The 2:1 ratio was chosen to ensure an adequate number of participants in the email intervention to form stair-walking groups together with colleagues. The examiner remained blinded to group allocation, and participants were instructed via email not to reveal their group allocation during follow-up examination. Author LLA performed the randomization and informed participants via email about group allocation.

### Primary Outcome: Aerobic Fitness

Aerobic fitness was estimated from a maximal cycle ergometer test, which has previously shown very good validity with direct measurements of oxygen uptake [20]. Men and women started the test with workloads of 105 and 70 watts, respectively, on a Monark cycle ergometer (model 874E, Monark AB, Stockholm, Sweden) and maintained a pedaling frequency of 70 revolutions per minute. The examiner added 0.5 kg resistance (35 watts) every other minute until the participant was not able to maintain a pedaling frequency of 70 revolutions per minute. The final workload (watts) and time using the final workload (seconds) determined maximal power output (MPO), calculated as MPO (watts) = final workload-35 + (35 × number of seconds at final workload/120). From this, the maximal oxygen uptake (VO_2max_) was estimated using the equation VO_2max_ (L/min) = 0.16 + (0.0117 × MPO) [20], and then divided by body weight (kg) and multiplied by 1000 to determine aerobic fitness (mL/min/kg).

### Subgroup With Low Aerobic Fitness

For exploratory subgroup analysis of employees with low aerobic fitness at baseline, participants were categorized according to the methods of Åstrand [21], defining women aged <30, 30-39, 40-49, 50-59, and >60 years with aerobic fitness of <35, <34, <32, <29, and <29 mL/min/kg, respectively, and men aged <30, 30-39, 40-49, 50-59, and >60 years with aerobic fitness of <44, <40, <36, <32, and <27 mL/min/kg, respectively, as having low aerobic fitness.

### Secondary Outcomes

Blood pressure (systolic/diastolic) was measured during the physical examination after 15 minutes rest. The examiner performed 3 measurements, and the average of these measurements was calculated. Participants were weighed on a Tanita scale (model Tanita Innerscan 543) providing information on body weight and body fat percentage. Height was measured with a Soehnle Foldable Ultrasound Height Rod. Body mass index (BMI) was calculated as BMI (kg/m^2^)=body weight/(height)^2^.

### Interventions

Before the study, a meeting was held with the management of the company who agreed to let employees in the email group walk the stairs for 10 minutes a day during work time. The management of the company felt that 10 minutes of group-based physical activities per day would not impair productivity and could perhaps contribute to improved social climate. Information about the aim and content of the project was posted on the intranet of the company. Further, the employees were invited via email to participate in an informational meeting about the project.

After baseline testing and randomization, participants in the email group were advised and reminded via email each Monday at 9 am over 10 weeks (ie, a total of 10 emails) to walk the stairs for 10 minutes a day during working days (typically 5 days a week). We chose this type of encouragement because emails are cheap, easy to administer, and have the ability to reach virtually all employees at office workplaces. Thus, even companies with limited resources would be able to implement such an intervention as opposed to physical activities requiring gyms, training equipment, and instructors. Furthermore, emails are the preferred source of information among employees [22]. However, no previous studies have documented the optimal frequency of emails to encourage physical activity. Whereas email overload may be a problem for some people [23], emails can be dealt with at one’s own convenience and, therefore, have limited impact on the normal workflow [24]. The frequency of once per week was chosen to not unnecessarily disturb the employees with too many emails, yet still frequently remind the employees to walk the stairs. This frequency of emails was based on experience from a previous workplace exercise study in office workers [25].

Participants in the email group were encouraged via email to form small groups with their colleagues in the same group and, if possible, to schedule the daily stair-walks. In detail, participants were advised to (1) walk at the same time each day to make it a habit, for example, before lunch or when meeting at work in the morning, (2) make an appointment in their work calendar (Outlook) with colleagues to use and improve social networks at the workplace, and (3) vary the stair-walks sometimes by changing the speed between flights of stairs and alternating between single and double steps to make it more challenging and avoid boredom. The content of the emails did not build on any specific behavioral theories, but varied slightly from week to week with the following 3 progression phases to ensure organization, intensity, and variation: (1) during the first weeks, the goal was to organize the stair-walking by scheduling and forming groups, (2) during the middle weeks, the goal was to increase exercise intensity by increasing the speed of stair-walking to ensure effectiveness, and (3) during the later weeks, the goal was also to introduce variation in the stair-walking (eg, varying between single and double steps) to avoid boredom.

**Figure 2:**
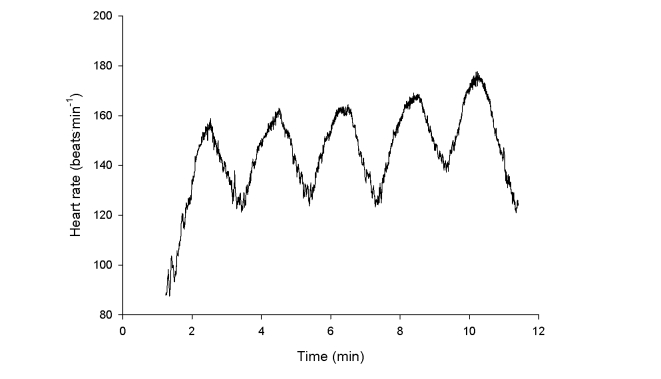
Heart rate recording from one of the participants during one of the daily stair-walks. This participant walked five times from the ground floor to the fourth floor of the building in 10 minutes.

The office building had 3 staircases with 3 to 4 floors. Stair-climbing is generally considered a vigorous type of physical activity [10]. In the present study, participants were offered to participate in heart rate measurements during stair-walking on the Wednesday of week 5 between 10 am and 12 am, during which time a research assistant presented with heart rate monitors at the main stair of the company. These measurements confirmed that the intensity of activity was high, with mean heart rate reaching 167 beats/min (SD 10) after 10 minutes of stair-walking (n=23), corresponding to 90% (SD 9%) of the heart rate reserve, calculated as (heart rate during stair-walk-resting heart rate)/(max heart rate-resting heart rate) × 100%. Figure 2 shows the recorded heart rate from 1 of the participants of the email group during 1 of the daily stair-walks. Participants in the control group were reminded each Monday at 9 am over 10 weeks via email to continue their usual physical activities during the project.

### Feasibility

After the first week of the intervention, the participants replied to an Internet-based questionnaire about sweating during stair-walking: “Did you experience sweating during the 10-minute stair-walks?” Participants had 3 response options: (1) no, (2) yes, but not to an extent that it bothered me, and (3) yes, to an extent that it bothered me. Participants also replied to an Internet-based questionnaire at 10-week follow-up. Adherence was evaluated by the question: “How many days per week during the last 10 weeks have you walked the stairs for at least 10 minutes at a time” (0, 1, 2,..., 7 days per week). Asking specific questions about adherence to workplace physical exercise in retrospect has shown good validity to day-to-day training diary registrations [26]. Participants also replied to the question: “During the last week, did you walk the stairs (1) together with colleagues, (2) alone, (3) alone and together with colleagues to an equal extent.” Multiple-choice questions concerning reasons for not participating as often as required (due to lack of time, lack of interest, illness, etc) were also given. Further, to determine the potential for long-term implementation of daily stair-walk, participants were asked whether they wished to continue daily stair-walking after termination of the research project, with the response options (1) yes, (2) yes, maybe, and (3) no.

### Other Physical Activities

We included questions to assess for initiation of other physical activities during the study period that could affect the primary outcome. At baseline and follow-up, participants filled in a modified version of the Saltin and Grimby [27] questionnaire concerning low-, medium-, and high-intensity leisure time physical activity. On an exploratory basis, we included this question as a covariate in the analyses on changes in fitness and blood pressure. Further, participants from both the control and email groups replied to the follow-up question regarding weekly frequency of 10-minute stair-walks.

### Statistics

The main outcomes were analyzed according to the intention-to-treat principle using a 2 × 2 mixed-factorial design, with time and group as independent categorical variables (fixed factors). The last-observation-carried-forward was not used because all methods of imputation have limitations. Instead, efforts were made at follow-up to explain to all participants, including dropouts, that their data were still required regardless of their level of actual participation, and we used the PROC GLIMMIX (general linear mixed models) of SAS version 9.2.( SAS Institute, Inc, Cary, NC, USA), which inherently accounts for missing values. Baseline values are reported as means (SD) and differences from baseline to follow-up as means (95% confidence interval).

## Results

### Adherence and Feasibility

In the email group, 82.7% (86/104) of the participants performed at least 3 stair-walking sessions per week. On average, the number of weekly stair-walking sessions was 3.3 (SD 1.3). During the last week of the intervention, participants from the email group replied that they had walked together with colleagues (65.3%, 68/104), alone (31.7%, 33/104), and alone and together with colleagues to an equal extent (2.9%, 3/104), respectively. Regarding the question after the first week of the intervention whether experiencing sweating during the 10-minute stair-walks, in the email group 5.2% (5/96) replied no, 71.8% (69/96) replied yes, but not to an extent that it bothered me, and 22.9% (22/96) replied yes, to an extent that it bothered me. In the email group, reasons for not participating as often as required were lack of time (51.0%, 53/104), illness (18.3%, 19/104), lack of interest (11.5%, 12/104), lack of motivation to start the program after vacation (7.7%, 8/104), difficulties in starting the program after illness (2.9% 3/104), lack of benefit from the stair-walk program (1.9%, 2/104), and lack of acceptance from nearest leader (1.0%, 1/104). None of the participants cited lack of acceptance from colleagues as a reason. In the email group, 26.2% (27/103) wished to continue stair-walking after the research project had ended, 53.4% (55/103) stated that maybe they wished to continue, and 20.4% (21/103) did not wish to continue stair-walking.

### Attrition

In the email group, 11 participants (10.4%) dropped out during the intervention period because of pregnancy (n=1), leaving the company (n=2), long-term vacation (n=1), stress (n=1), lack of time (n=2), and knee or ankle/foot injuries (n=4) of which 2 reported stair-walking as the cause of injury. In the control group, 5 participants (9.2%) dropped out during the intervention period because of long-term sickness absence (n=1) and with no reason given (n=4). Three and 2 of the dropouts from the email group and control group, respectively, presented for the follow-up examination, and 10 and 5, replied to the follow-up questionnaire, respectively. All dropouts were included in the intention-to-treat analyses.

### Intention-to-Treat Analysis

Table 1 shows baseline demographics of all participants of the 2 groups. Table 2 shows results from the intention-to-treat analysis for the change in fitness, blood pressure, and other variables from baseline to follow-up. A priori hypothesis testing showed a significant group × time interaction for aerobic fitness (*P*<.001). The email group improved aerobic fitness significantly more than the control group with a between-group difference of 1.45 mL/min/kg (95% CI 0.64-2.27). For the remainder of variables, there were no significant group × time interactions. We also performed exploratory analyses adjusting for the baseline level of physical activity on the changes in fitness and blood pressure. This had only minor influence on the results and the between-group difference for the change in fitness was 1.34 mL/min/kg (95% CI 0.51-2.16), and still nonsignificant for the changes in blood pressure.

### Subgroup With Low Aerobic Fitness

Table 3 shows baseline demographics of participants with low aerobic fitness of the 2 groups. Table 4 shows results from the subgroup analysis among individuals with low aerobic fitness at baseline for the change in fitness, blood pressure, and other variables from baseline to follow-up. There was also a significant group × time interaction for aerobic fitness (*P*=.008), and systolic (*P*=.03) and diastolic blood pressure (*P*=.04). There was also a tendency for the email group to decrease body weight (*P*=.08) compared with the control group, although this did not reach statistical significance.

### Other Physical Activities

The weekly duration of leisure time physical activity did not change significantly in any of the groups during the intervention period. Based on the follow-up questionnaire replies, 4 participants from the control group had performed 1 (n=2) and 2 (n=2) weekly sessions of 10-minute stair-walks.

### Test-Retest Reliability

Using the baseline and 10-week follow-up data from the control group (n=50), test–retest reliability was determined as the intraclass correlation coefficient (ICC) for the different outcomes, with ICC 0.98 for aerobic fitness, ICC 0.90 for diastolic and systolic blood pressure, and ICC 0.99 for weight, BMI, and body fat percentage.

**Table 1:** Baseline characteristics of participants in the email group and control group (N=160).

Variables		Email group (n=106)	Control group (n=54)
**Demographics**			
	Age (years), mean (SD)	42 (10)	43 (11)
	Height (cm), mean (SD)	171 (9)	172 (10)
	Weight (kg), mean (SD)	69 (14)	71 (11)
	Body mass index (kg/m^2^), mean (SD)	23 (4)	23 (3)
	Body fat %, mean (SD)	28 (8)	29 (9)
	Gender (women), n (%)	84 (79.2%)	41 (75.9%)
**Cardiovascular, mean (SD)**			
	Aerobic fitness (mL/min/kg)	36 (8)	38 (10)
	Systolic blood pressure (mmHg)	118 (14)	118 (14)
	Diastolic blood pressure (mmHg)	79 (9)	78 (8)
**Work-related, mean (SD)**			
	Sitting (% of work time)	89 (18)	90 (19)
	Weekly working hours	38 (3)	39 (4)
**Leisure time physical activity (hours/week), mean (SD)**			
	Low intensity	3.2 (1.7)	3.3 (1.7)
	Moderate intensity	1.8 (1.5)	1.9 (1.6)
	Vigorous intensity	0.3 (0.8)	0.6 (1.3)
**Other, n (%)**			
	Smokers	8 (7.5%)	6 (11.1%)

**Table 2:** Changes from baseline to 10-week follow-up (N=160).

Variables		Within-group differences from baseline to 10-week follow-up				Between-group differences from baseline to 10-week follow-up		
		Email group (n=106)		Control group (n=54)		Email vs Control		*P* value
		Mean	95% CI	Mean	95% CI	Mean	95% CI	
**Cardiovascular**								
	Aerobic fitness (mL/min/kg)	2.32	1.84, 2.79	0.86	0.20, 1.52	1.45	0.64, 2.27	<.001
	Systolic blood pressure (mmHg)	–2.62	–4.32, –0.93	–1.04	–3.36, 1.28	–1.58	–4.45, 1.28	.28
	Diastolic blood pressure (mmHg)	–2.85	–3.90, –1.81	–2.17	–3.61, –0.74	–0.68	–2.45, 1.10	.45
**Leisure time physical activity (hours/week)**								
	Low intensity	–0.15	–0.48, 0.18	–0.15	–0.61, 0.31	0.01	–0.57, 0.56	.98
	Moderate intensity	0.00	–0.30, 0.30	0.03	–0.38, 0.45	–0.04	–0.55, 0.47	.89
	Vigorous intensity	0.10	–0.05, 0.25	–0.04	–0.25, 0.17	0.13	–0.12, 0.39	.31
**Other**								
	Weight (kg)	–0.24	–0.60, 0.13	–0.12	–0.62, 0.38	–0.11	–0.73, 0.50	.72
	Body fat percentage (%)	–0.44	–0.71, –0.17	–0.47	–0.85, –0.10	0.03	–0.43, 0.49	.89

**Table 3:** Baseline characteristics of participants with low aerobic fitness in the email group and control group (n=56).

Variables		Email group (n=38)	Control group (n=18)
**Demographics**			
	Age (years), mean (SD)	44 (11)	46 (12)
	Height (cm), mean (SD)	170 (8)	166 (7)
	Weight (kg), mean (SD)	76 (14)	71 (10)
	Body mass index (kg/m^2^), mean (SD)	26 (5)	25 (4)
	Body fat %, mean (SD)	33 (7)	33 (8)
	Gender (women), n (%)	32 (84.2%)	15 (83.3%)
**Cardiovascular, mean (SD)**			
	Aerobic fitness(mL/min/kg)	28 (5)	28 (5)
	Systolic blood pressure (mmHg)	119 (17)	119 (14)
	Diastolic blood pressure (mmHg)	80 (10)	79 (8)
**Work-related, mean (SD)**			
	Sitting (% of work time)	85 (22)	86 (18)
	Weekly working hours	38 (3)	38 (3)
**Leisure time physical activity (hours/week), mean (SD)**			
	Low intensity	3.0 (2.0)	3.0 (2.0)
	Moderate intensity	1.0 (1.0)	1.0 (1.0)
	Vigorous intensity	0.0 (1.0)	0.0 (0.0)
**Other, n (%)**			
	Smokers	5 (13.2%)	4 (22.2%)

**Table 4:** Changes from baseline to 10-week follow-up in participants with low aerobic fitness at baseline (n=56).

Variables		Within-group differences from baseline to 10-week follow-up				Between-group differences from baseline to 10-week follow-up		
		Email group (n=38)		Control group (n=18)		Email vs Control		*P* value
		Mean	95% CI	Mean	95% CI	Mean	95% CI	
**Cardiovascular**								
	Aerobic fitness (mL/min/kg)	2.80	2.04, 3.62	0.95	–0.15, 2.05	1.89	0.53, 3.24	.008
	Systolic blood pressure (mmHg)	–3.19	–5.70, –0.68	1.63	–1.92, 5.17	–4.81	–9.16, –0.47	.03
	Diastolic blood pressure (mmHg)	–3.46	–4.99, –1.93	–0.79	–2.96, 1.37	–2.67	–5.32, –0.01	.04
**Leisure time physical activity (hours/week)**								
	Low intensity	0.11	–0.51, 0.72	0.00	–0.88, 0.88	0.11	–0.96, 1.18	.84
	Moderate intensity	0.16	–0.25, 0.58	–0.06	–0.65, 0.54	0.21	–0.51, 0.94	.55
	Vigorous intensity	0.00	–0.22, 0.22	–0.06	–0.37, 0.26	0.06	–0.32, 0.43	.77
**Other**								
	Weight (kg)	–0.81	–1.56, –0.07	0.33	–0.72, 1.38	–1.1	–2.43, 0.15	.08
	Body fat percentage (%)	–0.68	–1.16, –0.21	–0.14	–0.81, 0.54	–0.54	–1.37, 0.28	.19

## Discussion

### Principal Findings

In the present study, email-based encouragements to do 10 minutes of daily stair-walks together with colleagues resulted in 82.7% of the participants walking the stairs regularly, ie, at least 3 times per week. This resulted in improved aerobic fitness. This fairly high adherence shows that email-based encouragements to do daily stair-walks is a feasible way of implementing vigorous physical activity among employees in sedentary occupations.

The intention-to-treat analysis showed that aerobic fitness increased approximately 6% in the email group and 2% in the control group. Because aerobic fitness is an independent predictor of future cardiovascular disease [16,17], the results of the present study may be relevant from a preventive perspective. Although other studies using more comprehensive exercise protocols have found larger improvement in aerobic fitness [28], the simplicity and low cost of email-based encouragements to do daily stair-walks add positively to the arsenal of health-promoting initiatives to improve aerobic fitness of adults in sedentary occupations.

Most participants adhered to the email-based encouragement to do daily stair-walks, with 83% performing at least 3 sessions of 10 minutes per week, and a weekly average of more than 3 sessions, equivalent to 30 additional minutes of vigorous physical activity per week. The major reason for not participating as often as required was lack of time. Although this observation is consistent with Kruger et al’s [29] general observation concerning participation in health promotion programs, we have no underlying knowledge whether the reports reflect an actual lack of time or a more socially accepted way to express a disinterest or even laziness. Interestingly, few participants reported colleagues or the nearest leader as reasons for not participating. This seems to imply that important organizational circumstances were present and that the email-based encouragements to do daily stair-walks were generally accepted. Thus, a feeling of support from the employer may be important to successfully implement physical exercise by means of email-based encouragements at the workplace. The high acceptance level is also implied by the fact that almost 80% of the participants stated that they wished to continue with daily stair-walking after the project.

For health promotion, the ACSM and the AHA recommend moderate aerobic physical activity for at least 30 minutes on 5 days each week or vigorous aerobic physical activity for at least 20 minutes on 3 days each week [2]. However, many working-age adults lack the time for being physically active during leisure [7], and short bouts of physical activity at the workplace have been suggested as a solution [8]. The present study supports this notion and shows that email-based encouragements to do daily stair-walks at the workplace with colleagues can partly contribute toward reaching the recommended weekly dose of vigorous physical activity.

The participants in the subgroup with low fitness showed a 10% increase in aerobic fitness over 10 weeks of email-based encouragements to do daily stair-walks. This subgroup also showed a reduction in both systolic and diastolic blood pressure. Thus, the health benefits of email-based encouragements to increase physical activity appear to be higher among people with low fitness. Importantly, even small changes in blood pressure reduce the risk for cardiovascular mortality [30].

Adverse events in terms of knee or ankle/foot injuries directly related to the daily stair-walks were reported by 2 participants. Thus, a small risk for injury during stair-walks exists, which should also be considered by companies and employees when implementing such types of physical activity at the workplace. Nevertheless, stair-walking appears to be a relatively safe type of physical activity compared with different types of sports [31].

The intention of the weekly emails was to encourage short bouts of vigorous physical activity during the workday without the need for changing clothes and subsequent showering. Heart rate measurements clearly showed that the exercise intensity of stair-walking was high with heart rate reaching an average of 167 beats per minute. Approximately 3 of 4 participants (72%) did not experience sweating to a bothersome extent during stair-walking. However, only 5% did not experience sweating at all, whereas 23% experienced sweating to a bothersome extent. This may limit the feasibility of stair-walking at the workplace for approximately one-quarter of individuals because bothersome sweating may decrease motivation and require additional time for bathing. Moderate-intensity physical activities, for example, walking outside, which does not evoke sweating provides similar health-protective effects as more vigorous types of activity provided that the energy expenditure is equivalent [32]. Moderate-intensity activities, such as walking outside, although more time-consuming, may be preferred when sweating from vigorous activity is bothersome. Such activities can also be organized using Internet technology and reminder emails for the mutual benefit of employees and employers.

In the email group, 26% replied yes, 53% replied yes, maybe, and 20% replied no to the question about whether they wished to continue daily stair-walking after the research project. This indicates a certain potential for long-term sustainability of email-reminders to stimulate brief group-based physical activities at the workplace, but also shows that not all employees find stair-walking an attractive solution. Preferences and available time should be considered when implementing health-promoting physical activities at the workplace.

### Strengths and Limitations

There are both strength and limitations of the present study. The simplicity and low cost of email-based encouragements to form groups with colleagues to do physical activity strengthens the potential for wide-scale implementation of the research results at other companies. The randomized controlled design, blinding of examiners, the high follow-up percentage, and the low level of initiation of other physical activities during the intervention period improves the validity of the present results. The test methods were highly reliable over a 10-week period, allowing for very precise estimates of the intervention. However, because of the study design, participants were not blinded. Also, the risk of cross-contamination exists because all participants were from the same company. Based on the follow-up questionnaire, 7% of the participants from the control group participated in the 10-minute stair-walks, which may have slightly decreased between-group differences. Self-selection, ie, due to ethical reasons only volunteers are included, is a general limitation of randomized controlled trials. Thus, adherence may have been lower at the company level if all employees were included. Further, because of the email-based encouragement to form groups to do stair-walks, unknown cluster effects may have occurred. Further, not all office companies have accessible stairways, limiting the generalizability of the present findings to companies with accessible stairways. Finally, a limitation may be that the present study focused primarily on vigorous physical activity in terms of email-based encouragements to do stair-walks, and not specifically on breaking sedentary behavior several times during the working day. Future studies should combine both aspects (ie, reducing sitting time and increasing physical activity in sedentary populations) because they present distinct risk factors for adverse health outcomes [33]. Web or mobile applications to remind the employees to break sedentary behavior after a certain period, for example, every 30 minutes of continuous keyboard typing or mouse use, may be developed and tested in randomized controlled trials. Another target for future studies may be to investigate the effect of different theory-based motivators toward stair-walking (self-efficacy, goal setting, action planning, etc). This could be combined with research in delivering the encouragements in different ways (eg, emails, websites, text messages, smartphones) or with different frequencies (eg, daily, twice weekly, once weekly). Another possibility could be to develop smartphone applications interacting with the built-in 3D accelerometer of the phone to activate the buzz function after a certain period of inactivity to remind the employee to break sedentarism.

### Conclusions

In conclusion, email-based encouragements to do 10 minutes of daily stair-walks together with colleagues improve aerobic fitness among office workers. The high adherence shows that email-based encouragements to do daily stair-walks is a feasible way of implementing vigorous physical activity among employees in sedentary occupations. The weekly duration of stair-walking performed in the present study corresponds to approximately half of the ACSM and AHA recommended weekly amounts of vigorous physical activity.

## Multimedia Appendix 1

CONSORT-EHEALTH V1.6.2 checklist [19].

## Abbreviations

ACSM: American College of Sports Medicine
AHA: American Heart Association
BMI: body mass index
ICC: intraclass correlation coefficient
MPO: maximal power output
VO_2max_: maximal oxygen uptake
