# Supplementary material for: Cardiovascular Health Effects of Internet-Based Encouragements to Do Daily Workplace Stair-Walks: Randomized Controlled Trial
Source: J Med Internet Res. 2013 Jun 21;15(6):e127. doi: 10.2196/jmir.2340 (PMC3713894; doi:10.2196/jmir.2340)
Supplement: Supplementary file 1 [file jmir_v15i6e127_app1.pdf]

CONSORT-EHEALTH Checklist V1.6.2 Report

(based on CONSORT-EHEALTH V1.6), available at [http://tinyurl.com/consort-ehealth-v1-6].

2340

Date completed

5/30/2013 17:27:07

by

Lars L. Andersen

Cardiovascular health effects of email-based encouragements to do daily stair walk at the workplace: Randomized controlled trial

TITLE

1a-i) Identify the mode of delivery in the title

"internet-based encouragements"

1a-ii) Non-web-based components or important co-interventions in title

"internet-based encouragements"

1a-iii) Primary condition or target group in the title

"at the workplace"

ABSTRACT

1b-i) Key features/functionalities/components of the intervention and comparator in the METHODS section of the ABSTRACT

"Participants were randomly assigned (2:1 ratio) to an email group receiving weekly email-based encouragements to walk the stairs for 10 minutes a day or to a control group"

1b-ii) Level of human involvement in the METHODS section of the ABSTRACT

"Participants were 160 office workers (125/35 women/men, mean age 42 years"

1b-iii) Open vs. closed, web-based (self-assessment) vs. face-to-face assessments in the METHODS section of the ABSTRACT

"The examiner was blinded to group allocation."
"receiving weekly email-based encouragements "

1b-iv) RESULTS section in abstract must contain use data

"Adherence to the email encouragements was fairly high with 83% of the participants performing at least 3 sessions of 10-minute stair walks per week (mean 3.3 (SD 1.3) times per week). "

1b-v) CONCLUSIONS/DISCUSSION in abstract for negative trials

Not a negative trial

INTRODUCTION

2a-i) Problem and the type of system/solution

"The workplace may provide an optimal setting to encourage a healthier lifestyle as most adults spend the majority of their waking hours at work together with colleagues of which many have similar needs for physical activity [5]. Furthermore, in modern society most adult workers have the opportunity to communicate and organize physical activities through emails or internet-based media. In addition, overcoming the motivational barriers for performing regular physical activity is easier in a social setting such as the workplace than individually [6]. Internet technology helps create social networks, and social media may thus be used as a community setting at workplaces to encourage a healthier lifestyle and motivate physical activities. However, the need for special facilities and subsequent showering limits the feasibility of prolonged and strenuous physical exercise programs at the workplace. Further, lack of time is often cited as the major reason among adults for not being physically active [7]. Thus, short bouts of vigorous physical activity at the workplace that can easily be organized using internet technology and without the need for special facilities and subsequent showering may be preferred [8]. Robroek and coworkers showed that employees receiving monthly email prompts were 6 times more likely than those not receiving email prompts to continue using an internet-based physical activity and healthy nutrition program at the workplace [9]. Thus, emails and internet-based media may be an efficient and inexpensive method to organize and motivate participation in such physical activities. "

2a-ii) Scientific background, rationale: What is known about the (type of) system

"Robroek and coworkers showed that employees receiving monthly email prompts were 6 times more likely than those not receiving email prompts to continue using an internet-based physical activity and healthy nutrition program at the workplace [9]"

METHODS

3a) CONSORT: Description of trial design (such as parallel, factorial) including allocation ratio

"It was hypothesized that email-based encouragements to do daily stair walking improves aerobic fitness (primary outcome)."

3b) CONSORT: Important changes to methods after trial commencement (such as eligibility criteria), with reasons

No changes

3b-i) Bug fixes, Downtimes, Content Changes

Email were used in this study, typically not problematic

4a) CONSORT: Eligibility criteria for participants

"to 468 office workers in a large administrative company (i.e. a single workplace), and 345 replied. The inclusion criterion was willingness to participate in the study (N = 199). Exclusion criteria were: (i) a medical history of life threatening disease (N=8), (ii) pregnancy (N=2), (iii) unavailable during the study period (N=3), (iv) and blood pressure above 160/100 (determined later during physical examination). An invitation for a physical examination was sent to the remaining 186 employees, and 161 showed up. During the physical examination one employee was excluded due to blood pressure above 160/100. "

4a-i) Computer / Internet literacy

"All participants had computer / internet literacy"

4a-ii) Open vs. closed, web-based vs. face-to-face assessments:

"A screening questionnaire on health, physical activity and working conditions went out by email to 468 office workers in a large administrative company (i.e. a single workplace), and 345 replied"

"An invitation for a physical examination was sent to the remaining 186 employees, and 161 showed up."

4a-iii) Information giving during recruitment

"All participants gave written informed consent in agreement with the Declaration of Helsinki. "

4b) CONSORT: Settings and locations where the data were collected

"A randomized controlled trial was performed in Copenhagen, Denmark from February-June 2011. "

4b-i) Report if outcomes were (self-)assessed through online questionnaires

Outcomes were objectively measured

4b-ii) Report how institutional affiliations are displayed

5) CONSORT: Describe the interventions for each group with sufficient details to allow replication, including how and when they were actually administered

5-i) Mention names, credential, affiliations of the developers, sponsors, and owners

5-ii) Describe the history/development process

5-iii) Revisions and updating

5-iv) Quality assurance methods

5-v) Ensure replicability by publishing the source code, and/or providing screenshots/screen-capture video, and/or providing flowcharts of the algorithms used

5-vi) Digital preservation

5-vii) Access

"After baseline testing and randomization, participants in the email group were advised and reminded via email each Monday at 9 AM during 10 weeks (i.e. a total of 10 emails) to walk the stairs for 10 minutes a day during working days (typically 5 days a week). "

5-viii) Mode of delivery, features/functionalities/components of the intervention and comparator, and the theoretical framework

"Participants in the email group were encouraged via email to form small groups with their colleagues of the same group and if possible to schedule the daily stair walks. In detail, participants were advised to 1) walk on the same time each day to thereby make it a habit, e.g. before lunch or when meeting at work in the morning, 2) to make an appointment in their work calendar (outlook) with colleagues to thereby use and improve social networks at the workplace, 3) to sometimes vary the stair walks by changing the speed between flights of stairs and alternating between single and double steps to thereby make it more challenging and thus avoid boredom. The content of the emails did not build on any specific behavioral theories, but varied slightly from week to week with the following three progression phases to ensure organization, intensity and variation:

1)during the first weeks the goal was to organize the stair walking by scheduling and forming groups,

2)during the middle weeks the goal was to increase exercise intensity by increasing the speed of stair walking to ensure effectiveness,

3)during the later weeks the goal was also to introduce variation in the stair walking, (e.g. varying between single and double steps) to avoid boredom.

"

5-ix) Describe use parameters

5-x) Clarify the level of human involvement

5-xi) Report any prompts/reminders used

"the email group were advised and reminded via email each Monday at 9 AM during 10 weeks (i.e. a total of 10 emails) "

5-xii) Describe any co-interventions (incl. training/support)

"We included questions to assess for initiation of other physical activities during the study period that could affect the primary outcome. At baseline and follow-up, participants filled in a modified version of the Saltin & Grimby questionnaire concerning low-, medium-, and high-intensity leisure time physical activity [27]. On an exploratory basis we included this question as a covariate in the analyses on changes in fitness and blood pressure. Further, participants from both the control and email groups replied to the follow-up question regarding weekly frequency of 10-minute stair walks. "

6a) CONSORT: Completely defined pre-specified primary and secondary outcome measures, including how and when they were assessed

"Primary outcome: aerobic fitness

Aerobic fitness was estimated from a maximal cycle ergometer test, which has previously shown very good validity with direct measurements of oxygen uptake [20]. Men and women started the test with workloads of 105 and 70 watts, respectively, on a Monark cycle ergometer (model 874E, Monark AB, Stockholm, Sweden) and maintained a pedaling frequency of 70 revolutions per minute. The examiner added 0.5 kg resistance (35 watts) every other minute until the participant was not able to maintain a pedaling frequency of 70 revolutions per minute. The final workload (watts) and time using the final workload (seconds) determined maximal power output (MPO [watts] = final workload – 35 + (35 x time at final workload / 120)), from which the maximal oxygen uptake (VO2max) was estimated (VO2max = 0.16 + (0.0117 x MPO) [20], and divided by bodyweight to determine aerobic fitness (ml O2.min-1.kg-1).

"

"Secondary outcomes

Blood pressure (systolic / diastolic) was measured during the physical examination after 15 minutes rest. The examiner performed three measurements, and subsequently the average of these measurements was calculated. Participants were weighed on a Tanita scale (model Tanita Innerscan 543) providing information on bodyweight and bodyfat percentage. Height was measured with a Soehnle Foldable Ultrasound Height Rod. Bodymass index (BMI, unit: kg.m-2) was calculated as bodyweight divided by height*height.

"

6a-i) Online questionnaires: describe if they were validated for online use and apply CHERRIES items to describe how the questionnaires were designed/deployed

6a-ii) Describe whether and how "use" (including intensity of use/dosage) was defined/measured/monitored

"Adherence was evaluated by the question "How many days per week during the last 10 weeks have you walked the stairs for at least 10 minutes at a time" (0, 1, 2, …, 7 days per week). "

6a-iii) Describe whether, how, and when qualitative feedback from participants was obtained

"Feasibility

After the first week of the intervention the participants replied to an internet-based questionnaire on sweating during stair walking "Did you experience sweating during the 10-minute stair walks?" with three response options "no", "yes, but not to an extent that it bothered me", and "yes, to an extent that it bothered me". Participants also replied to an internet-based questionnaire at 10-week follow-up. Adherence was evaluated by the question "How many days per week during the last 10 weeks have you walked the stairs for at least 10 minutes at a time" (0, 1, 2, …, 7 days per week). Asking specific questions about adherence to workplace physical exercise in retrospect has shown good validity to day-to-day training diary registrations [26]. Participants also replied to the question "During the last week, did you walk the stairs 1) together with colleagues, 2) alone, 3) alone and together with colleagues to an equal extent". Multiple-choice questions concerning reasons for not participating as often as required ('due to lack of time', 'lack of interest', 'illness' etc) were also given. Further, to determine the potential for long-term implementation of daily stair walk, participants were asked whether they wished to continue daily stair walking after termination of the research project, with the response options 'yes', 'yes, maybe', and 'no'.

"

6b) CONSORT: Any changes to trial outcomes after the trial commenced, with reasons
"primary outcome were pre-defined. There were no changes in outcomes after the trial commenced. "
7a) CONSORT: How sample size was determined
7a-i) Describe whether and how expected attrition was taken into account when calculating the sample size

7b) CONSORT: When applicable, explanation of any interim analyses and stopping guidelines
not applicable
8a) CONSORT: Method used to generate the random allocation sequence
"Using a computer generated random numbers table"
8b) CONSORT: Type of randomisation; details of any restriction (such as blocking and block size)
"Simple randomization was used."
9) CONSORT: Mechanism used to implement the random allocation sequence (such as sequentially numbered containers), describing any steps taken to conceal the sequence until interventions were assigned
"The examiner remained blinded to group allocation, and participants were instructed via email not to reveal their group allocation during follow-up examination. "
10) CONSORT: Who generated the random allocation sequence, who enrolled participants, and who assigned participants to interventions
"Author LLA performed the randomization and informed participants via email about group allocation."
11a) CONSORT: Blinding - If done, who was blinded after assignment to interventions (for example, participants, care providers, those assessing outcomes) and how
11a-i) Specify who was blinded, and who wasn't
"The examiner remained blinded to group allocation, and participants were instructed via email not to reveal their group allocation during follow-up examination"
11a-ii) Discuss e.g., whether participants knew which intervention was the "intervention of interest" and which one was the "comparator"

11b) CONSORT: If relevant, description of the similarity of interventions
not applicable
12a) CONSORT: Statistical methods used to compare groups for primary and secondary outcomes
"The main outcomes were analyzed according to the intention-to-treat principle using a 2 x 2 mixed factorial design, with time and group as independent categorical variables (fixed factors). "
12a-i) Imputation techniques to deal with attrition / missing values
"The last-observation-carried-forward was not used as all methods of imputation have limitations. Instead, an effort was made at follow-up in explaining to all participants, including dropouts, that their data were still required regardless of their level of actual participation, and used the PROC GLIMMIX (general linear mixed models) of SAS version 9.2.(SAS institute, Cary, NC), which inherently accounts for missing values. "
12b) CONSORT: Methods for additional analyses, such as subgroup analyses and adjusted analyses
"Subgroup with low aerobic fitness
Table 3 shows baseline demographics of participants with low aerobic fitness of the two groups. Table 4 shows results from the subgroup analysis among individuals with low aerobic fitness at baseline for the change in fitness, blood pressure and other variables from baseline to follow-up"

RESULTS

13a) CONSORT: For each group, the numbers of participants who were randomly assigned, received intended treatment, and were analysed for the primary outcome
"Fig. 1. CONSORT flow diagram of participants through the study"
13b) CONSORT: For each group, losses and exclusions after randomisation, together with reasons
"Fig. 1. CONSORT flow diagram of participants through the study"
13b-i) Attrition diagram

14a) CONSORT: Dates defining the periods of recruitment and follow-up
"A randomized controlled trial was performed in Copenhagen, Denmark from February-June 2011. "
14a-i) Indicate if critical "secular events" fell into the study period

14b) CONSORT: Why the trial ended or was stopped (early)
not applicable
15) CONSORT: A table showing baseline demographic and clinical characteristics for each group
"Table 1. Baseline characteristics of participants in the email group and control group."
15-i) Report demographics associated with digital divide issues
"Table 1. Baseline characteristics of participants in the email group and control group."
16a) CONSORT: For each group, number of participants (denominator) included in each analysis and whether the analysis was by original assigned groups
16-i) Report multiple "denominators" and provide definitions
"Fig. 1. CONSORT flow diagram of participants through the study"
"analyzed according to the intention-to-treat principle "
16-ii) Primary analysis should be intent-to-treat
"analyzed according to the intention-to-treat principle "
17a) CONSORT: For each primary and secondary outcome, results for each group, and the estimated effect size and its precision (such as 95% confidence interval)
"Table 2. Changes from baseline to 10-week follow-up."
17a-i) Presentation of process outcomes such as metrics of use and intensity of use

17b) CONSORT: For binary outcomes, presentation of both absolute and relative effect sizes is recommended
not applicable
18) CONSORT: Results of any other analyses performed, including subgroup analyses and adjusted analyses, distinguishing pre-specified from exploratory

"We also performed exploratory analyses adjusting for the baseline level of physical activity on the changes in fitness and blood pressure. This had only minor influence on the results and the between-group difference for the change in fitness was 1.34 [0.51 to 2.16] ml O2.min-1.kg-1), and still non-significant for the changes in blood pressure. "

18-i) Subgroup analysis of comparing only users

19) CONSORT: All important harms or unintended effects in each group

"Adverse events in terms of knee or ankle/foot injuries directly related to the daily stair walks were reported by two participants. Thus, a small risk for injury during stair walk exists, which should also be considered by companies and employees when implementing such types of physical activity at the workplace. Nevertheless, stair walking appears to be a relatively safe type of physical activity compared with different types of sports [31]."

19-i) Include privacy breaches, technical problems

19-ii) Include qualitative feedback from participants or observations from staff/researchers

DISCUSSION

20) CONSORT: Trial limitations, addressing sources of potential bias, imprecision, multiplicity of analyses

20-i) Typical limitations in ehealth trials

"However, due to the study design participants were not blinded. Also, because all participants were from the same company the risk of cross-contamination exists. Based on the follow-up questionnaire 7% of the participants from control group had participated in the 10-minute stair walks, which may have slightly decreased between-group differences. Self-selection, i.e. due to ethical reasons only volunteers are included, is a general limitation of randomized controlled trials. Thus, adherence may have been lower at the company level if all employees were included. Further, due to the email-based encouragement to form groups to do stair walk group unknown cluster effects may have occurred. Further, not all office companies have accessible stairways, limiting the generalizability of the present findings to companies with accessible stairways. Finally, a limitation may be that the present study focused mainly on vigorous physical activity in terms of email-based encouragements to do stair walk, and not specifically on breaking sedentary behavior several times during the working day."

21) CONSORT: Generalisability (external validity, applicability) of the trial findings

21-i) Generalizability to other populations

"The simplicity and low cost of email-based encouragements to form groups with colleagues to do physical activity strengthens the potential for wide-scale implementation of the research results at other companies. "

21-ii) Discuss if there were elements in the RCT that would be different in a routine application setting

22) CONSORT: Interpretation consistent with results, balancing benefits and harms, and considering other relevant evidence

22-i) Restate study questions and summarize the answers suggested by the data, starting with primary outcomes and process outcomes (use)

"In the present study, email-based encouragements to do 10 minutes of daily stair walk together with colleagues resulted in 83% of the participants walking the stairs regularly, i.e. at least 3 times per week. This resulted in improved aerobic fitness. "

22-ii) Highlight unanswered new questions, suggest future research

"Future studies should combine both aspects – i.e. reducing sitting time and increasing physical activity in sedentary populations - as they present distinct risk factors for adverse health outcomes [33]. Web or mobile applications to remind the employees to break sedentary behavior after a certain period, e.g. every 30 minutes of continuous keyboard typing or mouse use, may be developed and tested in randomized controlled trials. Another target for future studies may be to investigate the effect of different theory-based motivators toward stair walking (self-efficacy, goal setting, action planning etc). This could be combined with research in delivering the encouragements in different ways (e.g. emails, websites, text messages, smartphones) or with different frequencies (e.g. daily, twice weekly, once weekly). Another possibility could be to develop smart phone applications interacting with the built-in 3D accelerometer of the phone to activate the buzz function after a certain period of inactivity to remind the employee to break sedentarism. "

Other information

23) CONSORT: Registration number and name of trial registry

"www.clinicaltrials.gov, number NCT01293253"

24) CONSORT: Where the full trial protocol can be accessed, if available

not applicable

25) CONSORT: Sources of funding and other support (such as supply of drugs), role of funders

not applicable

X26-i) Comment on ethics committee approval

x26-ii) Outline informed consent procedures

X26-iii) Safety and security procedures

X27-i) State the relation of the study team towards the system being evaluated
